# Differences Between Subclinical Ruminators and Reflectors in Narrating Autobiographical Memories: Innovative Moments and Autobiographical Reasoning

**DOI:** 10.3389/fpsyg.2021.624644

**Published:** 2021-03-02

**Authors:** Tilmann Habermas, Iris Delarue, Pia Eiswirth, Sarah Glanz, Christin Krämer, Axel Landertinger, Michelle Krainhöfner, João Batista, Miguel M. Gonçalves

**Affiliations:** ^1^Department of Psychology, Goethe University Frankfurt, Frankfurt, Germany; ^2^Psychology Research Centre, School of Psychology, Universidade de Minho, Braga, Portugal

**Keywords:** innovative moments, autobiographical reasoning, rumination, autobiographical memory, narrative

## Abstract

Reasoning may help solving problems and understanding personal experiences. Ruminative reasoning, however, is inconclusive, repetitive, and usually regards negative thoughts. We asked how reasoning as manifested in oral autobiographical narratives might differ when it is ruminative versus when it is adaptive by comparing two constructs from the fields of psychotherapy research and narrative research that are potentially beneficial: innovative moments (IMs) and autobiographical reasoning (AR). IMs captures statements in that elaborate on changes regarding an earlier personal previous problem of the narrator, and AR capture the connecting of past events with other parts of the narrator’s life or enduring aspects of the narrator. A total of *N* = 94 university students had been selected from 492 students to differ maximally on trait rumination and trait adaptive reflection, and were grouped as ruminators (*N* = 38), reflectors (*N* = 37), and a group with little ruminative and reflective tendencies (“unconcerned,” *N* = 19). Participants narrated three negative personal experiences (disappointing oneself, harming someone, and being rejected) and two self-related experiences of more mixed valence (turning point and lesson learnt). Reflectors used more IMs and more negative than positive autobiographical arguments (AAs), but not more overall AAs than ruminators. Group differences were not moderated by the valence of memories, and groups did not differ in the positive effect of narrating on mood. Trait depression/anxiety was predicted negatively by IMs and positively by AAs. Thus, IMs are typical for reflectors but not ruminators, whereas the construct of AR appears to capture reasoning processes irrespective of their ruminative versus adaptive uses.

## Introduction

Narrating an experience may help come to terms with it emotionally, cognitively, and socially (Habermas, 2019; Pennebaker and Seagal, 1999). Narrating problematic personal experiences may help to re-experience events and the related emotions and to organize and understand them (Smorti and Fioretti, 2016) also by sharing them with others (Rimé, 2020).

The cognitive function of narrating is supported by reasoning processes manifested in the use of arguments. In this paper we relate three ways of conceptualizing such reasoning processes which to date have been studied in different fields of psychology. In psychotherapy research, Gonçalves et al. (2011, 2017) traced processes of therapeutic change in session transcripts in terms of statements that contain exceptions (i.e., changes) to the problematic patterns of meaning that are typical of psychopathology; they termed these exceptions innovative moments (IMs; Batista et al., 2020). The authors proposed that IMs are the building blocks of more adaptive interpretations which emerge in successful psychotherapy. In narrative psychology, Habermas (2011) defined autobiographical reasoning (AR) as linking specific life events to other, distant events in life or to one’s personality and its development. Autobiographical reasoning may be used when narrating autobiographical memories and is an essential element in constructing a life story. Especially in times of change AR may be used to understand experiences and their impact by integrating them into the life story. IMs are thus statements about steps in the process of positive or adaptive change, whereas AR covers any kind of relation between local events and the rest of life or personality, independently of whether they refer to change or stability and of whether they are evaluated positively or negatively. Despite the differences between the two constructs, both IMs and AR refer to people’s efforts to understand life experiences.

A third way to conceptualize such reasoning processes besides IMs and AR is to differentiate between ruminative and reflective modes of thinking about life experiences. They have been studied with self-report measures, and, as far as we know, have never been studied from the perspective of IMs or AR. The purpose of this study is to relate these three conceptualizations of personal reasoning processes to each other by analyzing IMs and AR in narratives of personal experiences by ruminators and reflectors.

## Innovative Moments

Gonçalves et al. (2011, 2017) and Batista et al. (2020) developed a coding system to identify statements that indicate a change in the problematic patterns of meaning. It was first developed in the context of narrative psychotherapy (Matos et al., 2009), but was also applied successfully to emotion-focused (Mendes et al., 2011), client-centered (Gonçalves et al., 2012), cognitive-behavioral (Gonçalves et al., 2017), and constructivist grief psychotherapy (Alves et al., 2014).

In a bottom-up approach, markers that indicate change were identified and hierarchically organized into three levels of change. At level 1, problematic patterns of meaning are challenged by some form of elementary exception in which the person acts, feels, or thinks differently, not aligned with the problematic pattern. IMs at this level focus on creating some form of distance from the problematic pattern (e.g., “I’m starting to think differently than X,” “I need to do Y”; X being the problematic pattern and Y the exception).

Level 2 IMs emerge as change is described, usually by referring to how the person has changed positively, or the process that has been allowing change to occur (e.g., “Before I was X, now I’m Y”; X being the problematic pattern, and Y the innovation). Level 3 IMs – also termed reconceptualization – involves a meaningful articulation of the positive change in the self with the processes that, from the perspective of the person, allowed change to occur (e.g., “Before I was X, now I’m Y; because I’m doing Z”; X being the problematic pattern, Y the innovation, and Z the process of innovation). The coding of IMs in session transcripts involves first identifying the problems or maladaptive patterns, and then marking the different levels of IMs that represent exceptions to these problems.

In a series of studies that compared recovered with unchanged psychotherapy patients, higher level-IMs, but not lower level IMs emerged as a valid predictor of therapy success across several psychotherapy samples (e.g., Alves et al., 2014; Gonçalves et al., 2017). Moreover, level 2 and 3 IMs tended to precede symptom improvement (Gonçalves et al., 2017). For the purpose of this study we will consider level 1 IMs as low-level IMs (innovation that creates distance from the problem), and level 2 and level 3 as high-level IMs (innovation centered on change).

## Autobiographical Reasoning

Outside psychotherapy research, the processing of problematic experiences has been studied in narratives of autobiographical memories. When narrating experiences that are life-changing or discrepant with one’s view of oneself and the world, narrators may include attempts at rendering the experience compatible with their life story by relating it to other parts of life (e.g., “This special student culture at university made it so easy for me so that I made many, many friends; this was so different from my first weeks in grade school when I had been so afraid to speak to anyone”) and to the self-concept through AR (e.g., “These new experiences at university helped me become a less shy and more outgoing person”). Autobiographical reasoning is a specific form of interpreting or making meaning of life experiences. Authors of narrative studies in personality, developmental, and cognitive psychology tend to expect AR to be helpful for coping with negative events and therefore to correlate with well-being. Findings indirectly supporting a helpful role of reflecting on life are those by Pennebaker et al. (1997) that when repeatedly writing about potentially traumatic experiences, an increase in words indicating explanations predicted a reduction in the use of the health system later on. Also findings that narrating potentially traumatic experiences from a distanced present, rather than from an immersed past perspective is related to well-being (Kross and Ayduk, 2017) indirectly supports the positive role of AR which does require stepping back from the events to embed them in the life story. In their review, Adler et al. (2016) concluded that most narrative studies show a helpful role of AR (e.g., longitudinally, Mitchell et al., 2020).

However, there have also been findings of negative correlations between AR in narratives and well-being (cf. McLean and Mansfield, 2011). In some studies, such negative correlations showed in younger adolescents (Fivush et al., 2007; McLean et al., 2010; Chen et al., 2012; Waters and Fivush, 2015; Reese et al., 2017), suggesting that AR skills need to be well-developed before they might be used in a helpful way. However, even some studies with adults showed negative correlations between AR in narratives and concurrent well-being (e.g., Sales et al., 2013).

Therefore, several distinctions between kinds of AR that are related positively versus negatively with well-being and, inversely, symptoms of depression, have been suggested. First, autobiographical arguments (AAs) are defined formally as embedding an event in a life story. However, their content also appears to be important, for instance whether they make positive or negative statements about the narrator’s development. In a study of narratives of most negative and positive experiences, Lilgendahl and McAdams (2011) demonstrated that AR about positive personal growth correlated positively with well-being. Similarly, Banks and Salmon (2013) showed that in narratives of low and high points the relation between the frequency of AAs linking events to the narrator’s personality (self-event connections) and well-being depended on whether these arguments had positive or negative implications for the narrator (for example, “Living among these really poor people opened my eyes for how some people struggle. I have become a much less self-centered person since then”; cf. similarly Cox and McAdams, 2014; Merrill et al., 2016; Holm and Thomsen, 2018; Lilgendahl and McLean, 2020). In the same vein, another study found that victims of the Chilean dictatorship who intentionally reflected on their experience only showed post-traumatic growth if they also reframed it positively (Cárdenas Castro et al., 2019).

Second, Park (2010) suggested differentiating the activity of merely attempting to make meaning from successful meaning making – AR *per se* would count as attempt at meaning making. Initially coping efforts by attempting to make meaning may not yet be successful and be associated with negative affect, but later in the process the relationship may reverse as a new meaning is made and the event is understood better. Attempts at meaning making may be defined formally, but their success needs to be judged in terms of whether they are plausibly successful. Huang et al. (2020), for instance, found that in narratives about the loss of a parent both meaning made and AR with positive implications for the self correlated negatively with protracted grief; however, the mere frequency of self-event connections (a form of AR) and their relating to a change of personality predicted successful meaning making, but not protracted grief.

Third, increased AR may be most functional whenever there is a need for it, that is, when events are fresh and still need to be integrated into the life story, whereas habitually high levels of AR may be dysfunctional (Habermas and Köber, 2015a). For instance, only those participants who had experienced change in their lives in the past 4 years could buffer an ensuing sense of self-discontinuity through increased AR (cf. Tavernier and Willoughby, 2012; Habermas and Köber, 2015b). We now turn to a fourth specification of uses of AR related to differential associations with symptom and well-being, namely that between ruminative and solution-oriented, reflective uses.

## Rumination and Reflection

Rumination is defined as a maladaptive way of thinking about problems that is characterized by being inconclusive and repetitive or circular, involuntary or without reference to the situation at hand, and hence abstract and theoretical (e.g., Matthews and Wells, 2004). Rumination is involved in various psychological disorders. In obsessive-compulsive disorder rumination asks for reasons for things that are usually taken for granted (Griesinger, 1868). In depression, rumination focuses on one’s own bad character as evidenced in habitual ways of acting (repeated but not specific events; Watkins, 2008). In generalized anxiety disorder people are preoccupied with and worry about ill-defined and generalized bad things to come (Watkins, 2008; Newman et al., 2017). And in what has been termed embitterment, thinking is focused on perpetrators and revenge (Linden and Maercker, 2011). Rumination is generally a quality of thinking, but may also intrude communication with others. Thus, in turn, reflective processes that possess the opposite qualities of linearity and conclusiveness, being engaged intentionally and being motivated to solve a specific problem at hand may be adaptive in solving problems and assimilating or accommodating to new situations.

Habitual ruminative tendencies have most often been measured by self-report. Most self-report measures like the Response Styles Questionnaire (RSQ) focus on rumination in depression (e.g., Treynor et al., 2003), some also in anxiety disorders (Green et al., 2003). The Perseverative Thinking Questionnaire (PTQ; Ehring et al., 2011), in contrast, attempts to capture ruminative tendencies independently from their specific content. Some self-report instruments for self-reflection grew out of rumination measures for depression, such as the RSQ (Treynor et al., 2003), while others attempted to conceptualize reflection independently from specifically sad situations (Trapnell and Campbell, 1999).

Rumination *in actu*, the actual process of ruminating has been studied much less than the (self-reported) disposition for ruminating. Identifying characteristics of thinking in utterances is of high clinical validity, because clinicians know about what people think via what they say. For instance, a depressive explanatory style was identified in autobiographical memory narratives (Peterson et al., 1983; Adler et al., 2006; Habermas et al., 2008), which is both more objective and ecologically valid than self-report.

### The Present Study

To date, neither IMs nor AR have been studied in relation to habitual rumination and reflection. Also, IMs have rarely been studied outside the context of psychotherapy sessions. However, we expect IMs to be identifiable also in elicited narratives of autobiographical memories, because narratives are about events that happened out-of-the-ordinary, that breach canonical expectations (Bruner, 1990). With regard to habitual rumination versus reflection, we expect reflectors to use more IMs than ruminators, based on the idea that IMs indicate a more flexible thinking style. Based on the overall expectation that AR is helpful for coping with negative experiences, we also expect reflectors to use more AAs than ruminators (Hypothesis 1).

In addition, we tested the hypothesis that these differences show only in narratives of memories of negative experiences, because rumination focuses on negative affect, but not in memories that more inclusively reflect on positive and negative aspects of their identity (Hypothesis 2). In addition, we tested whether narrative characteristics (IMs and AR) predicted trait depression and anxiety over and above their prediction by trait rumination (Hypothesis 3). Finally, we tested whether the specific ways of narrating events affected narrators’ mood (Hypothesis 4).

## Pre-Study

### Participants and Procedure

Because trait rumination and trait reflection measured by questionnaires correlate highly with each other, we ran a pre-study for selecting extreme groups that clearly differed on both measures. In the context of large lecture courses students of law, economics, education, social sciences, and psychology were informed about the study. A total of 492 students (144 males) filled in three questionnaires and provided e-mail addresses.

### Material

#### Subscale Reflection of the Rumination-Reflection Questionnaire

The RRQ contains the subscales rumination and reflection to differentiate a depressive and a potentially healthy style of thinking about oneself (Trapnell and Campbell, 1999; German version Post, 2004; 12 items). We only used the reflection subscale (12 items) because we were less interested in the depressive content of rumination. It asks participants how much they enjoy thinking about abstract, philosophical questions.

#### Perseverative Thinking Questionnaire

The PTQ measures any kind of habitual ruminative thinking independent of sad, anxious, or angry content (Ehring et al., 2011; 15 items). The five aspects of rumination measured are the qualities of thinking being repetitive, intrusive, involuntary, inconclusive, and occupying attentional capacities. The scale specifically asks for the processing of particular experiences which makes it pertinent to our study.

#### Response Styles Questionnaire

The subscales Brooding and Reflective Pondering capture a maladaptive and a potentially adaptive form of thinking about sad experiences (Treynor et al., 2003; German version Huffziger and Kühner, 2012; 10 items).

### Results

Mean values for Reflection (RRQ-RF) were 42.29 (SD = 9.17), for PTQ 30.14 (SD = 9.93), for RSQ-Brooding 10.60 (SD = 3.08), and for RSQ-Reflection 10.12 (SD = 3.10). As expected, the two measures of rumination correlated highly (*r* = 0.60) as did the two measures of reflection (*r* = 0.43). However, correlations between rumination and reflection were also positive and not negligible: RSQ-RF correlated with PTQ, *r* = 0.45 and with RSQ-B, *r* = 0.40; while RRQ-RF correlated less with the two rumination scales, *r* = 0.23 and *r* = 0.14, respectively. We based the selection of groups on the reflection scale that correlated less with rumination (i.e., RRQ-RF) and the transdiagnostic rumination scale PTQ.

## Main Study

### Participants

To select three groups, one with high trait rumination and low reflection (“ruminators”), a second with high trait reflection and low rumination (“reflectors”), and a third low in both traits (“unconcerned”), for participation in the main study we approached individuals with maximal difference between the z-standardized scales PTQ and RRQ-RF, choosing a standard deviation of +1 or −1 of the difference as a cut-off, in addition to values above or below the respective mean on each scale. The unconcerned group was sampled to minimize the sum of the z-standardized values of both scales.

Starting with the most extreme values, we contacted participants of the pre-study to invite them to the main study. The final sample comprised 94 participants (69 women; mean age 21.8 years, SD = 3.7). We oversampled ruminators and reflectors because they were the focus of the study (Table 1). Groups differed significantly on the two scales used for the selection in the expected directions. However, Ruminators and Reflectors failed to differ on the reflection subscale of the RSQ. Also, men were underrepresented in the unconcerned group which unsurprisingly was the most difficult to motivate to participate (response rates of 19% in unresponsive, 54% in ruminators, and 57% in reflectors).

**TABLE 1:** Characteristics of ruminators, unconcerned, and reflectors.

	**Ruminators**		**Unconcerned**		**Reflectors**		**Group differences**	
**N women/men**	**28**	**10**	**16**	**3**	**25**	**12**		
	***M***	**SD**	***M***	**SD**	***M***	**SD**	***F* sign.**	**$\eta_{\text{p}}^{2}$**
Age	21.8	3.7	22.5	4.6	20.3	1.1	2.24	0.05
PTQ	44.6	5.8	18.6	5.9	24.2	6.1	Not tested	0.78
RRQ-RF	40.7	7.9	32.6	6.1	53.1	3.5	Not tested	0.64
RSQ-B	13.6	2.9	8.4	2.2	9.3	3.2	28.99***	0.39
RSQ-RF	11.7	2.4	7.3	1.9	10.3	2.6	21.14***	0.32
STADI trait	49.7	11.6	34.8	7.2	39.6	9.3	17.26***	0.28
LSS	23.7	5.7	28.5	2.9	26.5	6.8	4.87**	0.10
STADI state t_1_	39.8	9.9	33.1	5.5	35.2	7.0	5.38**	0.11
STADI state t_2_	21.0	5.7	16.8	3.6	18.3	3.3	6.61**	0.13
Difference t_2_−t_1_	18.8	7.3	16.3	2.9	16.9	6.3	1.36	0.03

df = 2, 91. PTQ, Perseverative Thinking Questionnaire; RRQ-RF, Subscale Reflection of Rumination-Reflection Questionnaire; RSQ-B, Subscale Brooding of Response Styles Questionnaire; RSQ-RF, Subscale Reflection of Response Styles Questionnaire; STADI, State-Trait Anxiety Depression; LSS, Life Satisfaction Scale.

**p < 0.01, ***p < 0.001.

### Procedure

Three to 12 weeks after the pre-study, participants were interviewed in a quiet office at the University by one of the second to fifth authors who at the time were Master students of Psychology, blind to participants’ group membership and about equally distributed across the three groups. Participants were informed about the main study and provided consent. They filled in a mood questionnaire, generated five memories, narrated them always in the same order, rated each on several scales, again filled in the mood questionnaire, and finally also filled in three other questionnaires. Interviewers maintained a friendly, non-judging attitude and encouraged participants in a natural, non-selective way. Interviews lasted about 1 h. Undergraduate students of Psychology obtained course credit. The study obtained approval by the Ethics Committee of the Faculty of Psychology and Sports of Goethe University Frankfurt (#2014-113).

### Material

#### Narratives

We asked participants first to select and write down a sentence each about five memories of specific events (<24 h) that were older than 6 months but not older than 5 years. We restricted the age of the memories to exclude both very recent ones that have not yet stabilized and very old ones that are usually self-defining and highly important in order to make memories more or less comparable, but we also chose a long enough period so as not to make it too difficult to find the requested kind of memory. We asked for two memories that are highly self-relevant and require change in the self, a “turning point event” (event 1) and “an event from which you learned a lesson” (event 5) – these events could vary in valence. We also asked for three negative events which might evoke ruminative thinking, varying in who was responsible and who was harmed: an experience of “being very disappointed by yourself” (event 2), of “having hurt someone” (event 3), and of “having been rejected or abandoned by someone” (event 4). To instigate AR, all narratives were followed up by the questions “What does the experience tell about yourself or your life? Which were the consequences for you and your life? How may the experience have changed you?” Narratives were tape-recorded, transcribed verbatim, and segmented into propositions (main and subordinate clauses).

#### Autobiographical Arguments

These comprise typical forms of biographical reasoning, that is, of embedding an event in the life story (Habermas and Bluck, 2000; Habermas and Paha, 2001; Habermas, 2011). We coded three groups of AAs: The first group comprises causal-motivational links between events and personality (Linde, 1993; Habermas and Bluck, 2000; Habermas and Paha, 2001), complemented and termed self-event connections by Pasupathi et al. (2007). Self-event connections may be of two types: change engendering or stability-maintaining. The first comprise events that changed personality and self-understanding (e.g., “since my parents divorced, I became a more shy and cautious person”); the latter comprise statements in which a personality trait explains an action or is illustrated by it, or in which the action is in stark contrast to the typical trait (e.g., “I didn’t ask her out because I am kind of a shy guy”). The second group of AAs comprises statements, either about stability or change, in how one views parts of life (termed change of view) regarding knowledge (e.g., “when my parents told me at age 45 that I had been adopted, my world was shaken”), regarding evaluation on the good-bad continuum (e.g., “It was a really difficult time for me, but now I think it was worth it because it taught me how to choose better”), and understanding (e.g., “Only when I became a father myself, did I understand why my father had acted like that”). In addition, we also coded whether the knowledge, evaluation, or understanding was evaluated as positive or negative at present. Thus, this group is about an increase in knowledge (or its stability) and reappraisals (or stable appraisals). The third group of AAs contained heterogeneous “other AA,” including contextualizing an event by reference to what is normal at a given age (“that’s what’s on your mind when your 13”), providing an individual biographical background that explains an individual’s actions (“when a car was approaching he really freaked out; later I learned that as a kid he had been run over by a car”), formative experiences (“this really left a strong imprint on me”), lessons learnt (“I learned that when I fall in love again, I should continue to work for school”), general insights (“probably you always stay attached to the one whom you kiss first”), and turning points (“this was really a break in my life”; cf. Köber et al., 2015). The first and third group of AAs were coded with established manuals (e.g., Köber et al., 2015), the second group with a new manual.

Codes were identified for each proposition. For each narrative, the number of codes was divided by the number of propositions and multiplied by 100, resulting in the percentage of propositions with any given code. Two raters trained coding until they were confident, and then coded 50 narratives by 10 participants independently to establish an initial interrater reliability; disagreements were settled by discussion. The remaining 420 narratives were divided between the coders; in addition, 30 narratives were coded by both coders unbeknownst to them, to establish a follow-up reliability. Reliabilities for self-event connections were *K* = 0.91 (follow-up reliability 0.75), for change of view *K* = 0.81 (0.81), and for their valence *K* = 0.78 (0.80), for other AAs *K* = 0.93 (0.72), evidencing mostly excellent to at least satisfactory reliabilities.

#### Innovative Moments

Innovative moments were identified on the basis of the IMCS (Version 8.0; Gonçalves et al., 2011). Two coders (PE and SG) were trained by an expert coder (JB) in a standardized five-step procedure with original material from psychotherapy sessions provided by the manual’s authors. Regarding the narratives of this study, the two coders, supervised by the expert coder, first identified for each narrative a problem, that is a difficulty that needs to be overcome. In a second step they marked beginning and endings of text segments containing IMs, that is statements about actions, emotions, thoughts, forms of relating that move away from the problem or identified some sort of change. In a third step they identified the specific level of IM for each text segment. Because the IMs coding schema had originally been devised for psychotherapy sessions, three rules were added to adapt the manual to non-therapeutic autobiographical narratives. First, if no problem was explicated, only low-level IMs could be coded; second, IMs could only be coded if the problem regarded the narrator’s self and not just an external problem; third, when narrators spoke about having learned something, IMs could only be coded if the learning had positive consequences.

Agreement regarding the percentage of words identified as segments containing IMs was determined twice. Narratives of the first 10 participants were coded independently by both coders, resulting in 94% agreement, and a Cohen’s Kappa for the coding of level of IMs of *K* = 0.89. The remaining narratives were divided equally between the two coders, and unbeknownst to them, narratives of six more participants were coded by both, resulting in 96% agreement on segments and a Kappa of *K* = 0.89 for the level of IMs. We used the percentage of text with IMs of each level as well as the sum of both as indicators of the presence of IMs.

#### Ratings of Memories

All memories were rated by participants for memory age, valence then and now, for having learnt a lesson (one item), and for frequency of remembering [three items: “Still today thoughts about the event come popping up,” “Still today I see the event before my inner eye,” and “I still frequently think of the event” (Cronbach’s α = 0.81)]. The two items each for positive and negative (inverted) valence then (*r* = 0.90) and today (*r* = 0.89), respectively, were combined to form indices of positivity then and now.

#### State Anxious and Depressed Mood

Mood was measured twice with the state scales of the STADI (Laux et al., 2013), a German sequel to the STAI (Spielberger et al., 1968) with scales for anxiety and depression (10 items each); we used the combined sum score as a global measure of mood.

#### Trait Anxiety and Depression

The trait version of the STADI (Laux et al., 2013) comprises 10 items each for depression and anxiety; we used the combined sum score as a global measure of habitual symptoms.

#### Life Satisfaction

Satisfaction was measured with the Life Satisfaction Scale (5 items; Diener et al., 1985; German version by Glaesmer et al., 2011).

### Results

The sample size was chosen for pragmatic reasons. *Post hoc* power analysis (with G^∗^power software^1^) showed that with a power of 0.80, effects needed a size of 0.33 to be detected. This means that this study only detected effects of mean to large size with sufficient probability, which is adequate because we studied extreme groups.

#### Descriptive Results

For descriptive purposes, we ran analyses of variance (ANOVAs) of measures of levels of depressive and anxiety symptoms and well-being with group as the only factor. Because the three groups had been created using values on the PTQ and RRQ, they differed on these as well as on the RSQ which also measures rumination and reflection. Ruminators were most depressed and least satisfied with their lives, reflectors next, and unconcerned the least depressed (Table 1).

Length of narratives and ratings of memories were tested for exploratory purposes with repeated analyses of variance (rANOVAs), with groups as between and memories as within factors. We report tests for overall group differences, planned contrasts between ruminators and reflectors as well as planned contrasts between the two self-related versus three negative memories. Groups did not differ in length of narratives. Ruminators had the highest values in rated frequency of remembering, followed by reflectors. Although groups did not differ in the past valence of events, ruminators rated them as most negative today, accordingly as having improved least, and there was a trend for ruminators to having learnt the least from them, while reflectors evidenced the opposite pattern. Testing differences between memories, the two self-relevant memories unsurprisingly were evaluated more positively than the three negative memories, both in the past and the present. They were also longer, remembered more frequently, and more lessons were learnt from them (Table 2).

**TABLE 2:** Narrative characteristics of ruminators, unconcerned, and reflectors, for two self-relevant memories (upper lines) and three negative memories (lower lines).

	**Ruminators**		**Unconcerned**		**Reflectors**		**Groups^1^ (2, 91)**		**Negative versus self memories^2^ (1, 91)**		Interaction memories* groups^3^ (2, 91)	
	***M***	**SD**	***M***	**SD**	***M***	**SD**	***F* sign $\eta_{\text{p}}^{2}$**		***F* sign $\eta_{\text{p}}^{2}$**		***F* sign $\eta_{\text{p}}^{2}$**	
N propositions	61.7	29.5	66.2	44.0	74.2	39.0	0.98	0.02	30.59***	0.25	0.47	0.01
	53.0	27.3	57.0	37.2	61.9	30.5						
**Mean ratings of five events (1–5)**												
Frequency of remembering	3.7	0.7	3.2	0.9	3.5	0.8	5.16**	0.10	31.50***	0.26	0.50	0.01
	*3.3*	*0.7*	*2. 7*	*0.7*	*2.9*	*0.7*						
Positivity then	3.0	1.3	2.8	1.2	3.2	0.5	0.74	0.02	143.01***	0.61	0.92	0.02
	*1.4*	*0.3*	*1.5*	*0.5*	*2.4*	*0.5*						
Positivity now	3.5	1.3	3.4	1.2	4.1	0.9	4.40*	0.09	99.39***	0.52	1.10	0.02
	*2.0*	*0.3*	*2.3*	*0.3*	*2.5*	*0.3*						
Positive change of valence	0.5	0.7	0.6	0.7	0.9	0.8	3.12*	0.06	3.21	0.03	0.08	0.00
	*0.7*	*0.3*	*0.3*	*0.8*	*2.0*	*0.8*						
Lesson learned	4.4	0.6	4.5	0.5	4.8	0.4	3.06	0.06	44.84***	0.33	0.29	0.01
	*3.9*	*0.8*	*4.0*	*0.6*	*4.2*	*0.7*						
**Autobiographical reasoning (% of propositions)**												
Other autobiographical arguments	4.2	2.2	3.8	2.7	4.0	1.4	0.28	0.00	79.79***	0.49	0.63	0.01
	*2.5*	*2.3*	*2.8*	*1.5*	*2.8*	*2.5*						
Self-event connections	1.5	1.4	1.2	1.4	1.1	1.2	0.92	0.02	4.85*	0.05	0.18	0.00
	*1.0*	*1.1*	*0.9*	*1.0*	*0.9*	*0.7*						
Change versus stability	1.2	1.3	0.6	1.7	0.6	1.2	0.84	0.02	40.72***	0.31	1.75	0.04
	*−0.4*	*0.8*	*−0.3*	*1.2*	*−0.4*	*0.8*						
Change of view	2.0	1.1	2.2	1.0	2.2	1.1	0.03	0.00	0.18	0.00	0.43	0.01
	*2.1*	2.2	*2.1*	2.2	*2.1*	2.2						
Change versus stability	2.0	1.2	2.2	1.0	2.2	1.2	0.08	0.00	0.92	0.01	0.15	0.00
	*2.0*	*1.2*	*2.0*	2*.3*	*2.0*	*1.2*						
Positivity	1.5	1.4	1.4	1.0	1.8	0.9	3.82*	0.08	5.12*	0.05	2.13	0.05
	*0.7*	*1.2*	*2.4*	*2.3*	*2.5*	*2.4*						
**Innovative moments**												
IM low	7.6	5.6	8.7	7.7	9.4	5.5	3.19*	0.07	26.37***	0.23	0.26	0.01
	*3.3*	*3.3*	*4.8*	*3.4*	*6. 6*	*4.7*						
IM high	13.0	12.3	9.6	9.4	15.2	13.2	4.40*	0.09	68.19***	0.43	1.94	0.04
	*0.0*	*0.0*	*2.3*	*2.9*	*4.9*	*4.8*						

*^∗^*p* < 0.05, ^∗∗^*p* < 0.01, ^∗∗∗^*p* < 0.001.*

*^1^Overall differences between three groups; planned contrast between ruminators and reflectors is reported in the text.*

*^2^Planned contrast between self-relevant and negative event narratives.*

*^3^Interaction between contrast between self-relevant and negative event narratives and three groups.*

Then we explored the correlations between IMs and AAs which partially overlap theoretically, because high-level IMs may involve insights and self-event connections. Table 3 presents correlations with values averaged across all five narratives (*N* = 94; upper right part of table) and at the level of narratives (*n* = 470; lower left part of table). To compare constructs, correlations at the level of narratives are more informative. When summing up all IMs, they correlated weakly, but in the expected direction, with the proportion of other AAs (especially learning a lesson, insight, and turning point; but not the proportion of self-event connections or change of view), with the change-relatedness of self-event connections (all four sub-indicators contributed to this correlation, but not of change of view), and with positive consequences of the change of view for the narrator. Thus, the two constructs, IMs and AA, mostly cover different phenomena; they do overlap in the area of positively evaluated change of self and self-understanding.

**TABLE 3:** Correlations between narrative measures and questionnaires (averaged across narratives in upper rectangle and upper right triangle, within narratives in lower left triangle and lower rectangle).

	**IM all**	**IM low**	**IM high**	**OAA**	**SEC**	**SECc**	**CoV**	**CoVc**	**CoVp**
Age	0.21	0.14	0.14	–0.27	–0.30	–0.01	–0.21	–0.22	–0.01
PTQ	–0.21	–0.16	–0.15	–0.02	0.11	0.13	–0.02	–0.04	–0.14
RRQ-RF	0.36	0.25	0.29	–0.01	–0.08	0.09	–0.02	0.01	0.15
RSQ-B	–0.21	–0.12	–0.18	0.08	0.10	0.11	0.14	0.15	–0.06
RSQ-RF	–0.01	–0.09	0.06	–0.01	0.11	0.19	0.01	0.02	–0.02
STADI trait	–0.23	–0.10	–0.19	0.22	0.11	0.09	0.17	0.18	0.03
LSS	0.23	0.01	0.22	0.05	–0.14	0.07	–0.03	0.01	0.13
ΔSTADIst	0.13	0.05	0.11	–0.13	0.01	0.04	–0.11	–0.10	–0.04
**Narrative measures:**									
IM all		0.39	0.78	0.08	–0.05	0.20	–0.07	–0.02	0.35
IM low	0.45		–0.21	0.24	0.00	0.33	0.27	0.27	0.41
IM high	0.81	–0.09		–0.09	–0.05	–0.04	–0.23	–0.18	0.12
OAA	0.19	0.17	0.09		0.38	0.44	0.65	0.64	0.45
SEC	0.02	0.00	0.03	0.18		0.25	0.32	0.30	0.12
SEC change	0.24	0.11	0.20	0.32	0.17		0.33	0.35	0.26
CoV	0.00	0.10	–0.08	0.28	0.09	0.14		0.97	0.55
CoV change	0.02	0.11	–0.06	0.28	0.09	0.13	0.94		0.52
CoVpos	0.29	0.30	0.12	0.24	–0.01	0.20	0.49	0.46	
**Memory ratings:**									
Pos. then	0.42	0.22	0.37	0.16	0.03	0.17	–0.13	–0.11	0.05
Pos. now	0.46	0.27	0.37	0.08	0.01	0.18	–0.09	–0.09	0.08
Pos. change	0.10	0.09	0.04	–0.08	–0.02	0.04	0.04	0.01	0.05
L. learnt	0.35	0.23	0.25	0.11	0.02	0.14	0.06	0.09	0.23
Memory age	0.01	–0.02	0.03	0.02	–0.04	–0.01	–0.03	–0.02	–0.04

*IM all, proportion of all innovative moments; IM low, proportion of problem distancing innovative moments; IM high, proportion of centering on change and reconceptualization innovative moments; OAA, % other autobiographical arguments; SEC, % self-event connections; SECch, change-related minus stability maintaining SECs; CoV, % change of view; CoVch, change-related minus stability maintaining CoVs; CoVp, CoVs with positive minus CoVs with negative consequences for narrator; PTQ, Perseverative Thinking Questionnaire; RRQ-RF, Subscale Reflection of Rumination-Reflection Questionnaire; RSQ-B, Subscale Brooding of Response Styles Questionnaire; RSQ-RF, Subscale Reflection of Response Styles Questionnaire; STADI, State-Trait Anxiety Depression; LSS, Life Satisfaction Scale; ΔSTADIst, difference between mood in STADI state at beginning minus at the end of the session; Pos. then, rating of positivity minus negativity then; Pos. now, rating of positivity minus negativity at present; Pos. change, positive change as difference between two preceding rating values; L. learnt, rating of lesson learnt. In the upper right triangle *r* > 0.20 *p* < 0.05, *r* > 0.26 *p* < 0.01, *r* > 0.35 *p* < 0.001.*

#### Hypothesis 1: Differences Between Ruminators and Reflectors in Autobiographical Reasoning and Innovative Moments

To test Hypothesis 1 that reflectors use more IMs and AAs than ruminators, and to limit the inflation of alpha-error, we first ran one repeated multivariate analysis of variance (rMANOVA) with group as independent factor and memories as within factor for each of the two groups of indicators. As expected, IMs differed between groups [Wilks’ lambda Ʌ = 0.81, *F*(4,180) = 5.04, *p* = 0.001, $\eta_{\text{p}}^{2}=0.10$], but AAs did not [Wilks’ lambda Ʌ = 0.84, *F*(12,172) = 1.31, *p* = 0.215, $\eta_{\text{p}}^{2}=0.08$].

Univariate follow-up tests for IMs showed that both low and high-level IMs differed significantly between groups. More specifically, in a planned contrast, reflectors had significantly more low-level IMs (contrast estimator = 2.31, *p* = 0.014) and high-level IMs (contrast estimator = 3.81, *p* = 0.007) than ruminators; the unconcerned group was in an intermediate position between ruminators and reflectors. Among the specific kinds of AAs, only the positivity of change of views differed between groups in the expected direction (see Table 2).

#### Hypothesis 2: Differences Between Self-Relevant and Negative Memories in Autobiographical Reasoning and Innovative Moments

Hypothesis 2 stating that differences between groups only show in reflective processes in narratives of negative memories was tested by the interaction term of the contrast between self-relevant versus negative memories with groups. Although the self-relevant memories contained more IMs, self-event-connections, and other AAs than negative memories (Table 2, 3rd and 4th-to last columns), the group differences did not differ between the two kinds of memories (Table 2, last two columns), clearly refuting Hypothesis 2.

#### Hypothesis 3: Prediction of Levels of Symptoms and Well-Being

Table 3 shows the correlations between narrative variables and levels of depressive and anxiety symptoms, well-being, and change in mood. To test Hypothesis 3 that IMs and AAs predict levels of symptoms and well-being, and that they do this over and above trait rumination, we ran a hierarchical multiple stepwise regression for each of the two dependent variables, potentially entering first narrative measures, and then the four rumination and reflection scales as additional possible predictors (Table 4). Zero-order correlations of PTQ, RRQ-RF, RSQ-B, and RSQ-RF with trait depression and anxiety (STADI) were *r* = 0.60, −0.01, 0.68, and 0.34, and with life satisfaction (LSS) *r* = −0.32, −0.03, −0.26, and −0.37. Trait depression and anxiety was predicted first negatively by overall IMs, and in addition positively by other AAs. When entering the rumination scales as predictors in further steps, both rumination scales added significantly to the prediction; AAs remained a significant predictor, whereas IMs no longer contributed significantly to the prediction (Table 4). Thus, IMs in narratives predicted lower trait depression and anxiety, whereas the frequency of AAs was unexpectedly related positively to trait depression and anxiety; only the prediction by AAs, but not by IMs added to a prediction by rumination.

**TABLE 4:** Hierarchical multiple regressions of narrative measures and trait rumination on trait depression and anxiety and on life satisfaction.

	**Model 1**				**Model 2**				**Model 3**				**Model 4**			
	***B***	***SE B***	**β**	**r*_p_***	***B***	***SE B***	**β**	**r*_p_***	***B***	***SE B***	**β**	**r*_p_***	***B***	***SB B***	**β**	**r*_p_***
**DV = STADI**																
IM all	−0.37	0.16	−0.23*	−0.23	−0.40	0.16	−0.25*	−0.26	−0.18	0.12	−0.12	−0.16	−0.16	0.12	−0.10	−0.14
OAA					1.84	0.76	0.24*	0.25	1.34	0.58	0.18*	0.24	1.49	0.57	0.19*	0.27
RSQ-B									2.01	0.24	0.64***	0.66	1.43	0.34	0.45***	0.41
PTQ													0.23	0.10	0.26*	0.25
*R*^2^ *adj*	*0.05*				*0.11*				*0.50*				*0.53*			
**DV = LSS**																
IM all	0.19	0.08	0.23*	0.23	0.13	0.08	0.16	0.17								
RSQ-B					−0.55	0.16	−0.34**	−0.34								
*R*^2^ *adj*	*0.05*				*0.25*											

*DV, dependent variable; IM all, proportion of all innovative moments; OAA, % other autobiographical arguments; RSQ-B, Subscale Brooding of Response Styles Questionnaire; PTQ, Perseverative Thinking Questionnaire; RSQ-RF, Subscale Reflection of Response Styles Questionnaire; STADI, State-Trait Anxiety Depression; LSS, Life Satisfaction Scale.*

*^∗^*p* < 0.05, ^∗∗^*p* < 0.01, ^∗∗∗^*p* < 0.001.*

Correlations of narrative measures and rumination with life satisfaction were generally lower than with levels of depressive and anxiety symptoms. Only IMs positively predicted life satisfaction, a relation that was no longer significant once rumination was entered in the equation (Table 4).

To explore whether the correlations were moderated by the kind of memory narrated, we compared correlations of IMs and AAs with levels of depressive and anxiety symptoms and life satisfaction separately for the three negative (IMs: *r* = −0.19, 0.15; AAs: *r* = 0.15, 0.05) and the two self-related memories (IMs: *r* = −0.19, 0.23; AAs: *r* = 0.21, 0.03), finding no substantial differences.

#### Hypothesis 4: Influences of Narrating on Mood

The general increase in mood from before to after narrating the five memories was not predicted by any of the narrative measures or any of the trait rumination and trait reflection measures.

## Discussion

The attempt to differentiate reasoning processes in autobiographical narratives by habitually more ruminative versus reflective narrators was only partially successful. The study tested the hypothesis that those with a maximal difference between dispositions to ruminate and to reflect adaptively would differ in the arguments they use in oral narratives about specific negative and self-relevant personal experiences in a non-clinical sample. In addition to AAs, which in the past had showed mixed correlations with well-being, we also studied IMs as an indicator of reasoning about positive personal change. Only IMs and the degree of positivity of AAs, but not the relative frequency of AAs, differed between ruminators and reflectors. Thus IM-statements involving positive changes in problematic experiences were more frequent in autobiographical narratives of people who habitually reflect than in narratives by people who habitually ruminate. This confirms that the construct of IM does generalize from psychotherapy sessions to narratives of everyday experiences in a non-clinical sample.

In contrast, the relative extent of AR, defined as any connecting of events with distant other life events or personality irrespective of the specific content of arguments used, did not differ between ruminators and reflectors. We believe this shows that AR may be used both either circularly, vaguely, and inconclusively, or productively leading to a successful integration of an event into a life. Only the positivity of AAs was higher in narratives of reflectors than in those by ruminators. Importantly, the findings confirm Lilgendahl and McAdams (2011) and others’ findings that only AAs with positive implications, but not the overall frequency of AAs correlates with well-being.

In addition to the mixed results regarding differences between ruminators and reflectors, the differences that we did find were not dependent on the negativity of the memory narrated. Moreover, the three clearly negative memories did not elicit proportionally more, but rather fewer arguments, indicating less reasoning intensity. This may indicate that the instructions for the two self-related memories specifically required reasoning to show that an event was indeed a turning point in one’s life path and that indeed lessons had been learned. The instructions for the negative experiences allowed for, but did not require reasoning process. Furthermore, in retrospect we realized that the three negative experiences we asked for were more specific than the two self-related memories, and therefore possibly less important, which might be an additional explanation for the more frequent reasoning in self-related than in negative memories. Possibly, the degree of resolution of negative events correlates with the frequency of IMs.

We related reasoning processes not only to ruminative versus reflective processes, but also to trait depression/anxiety and hedonistic well-being, to compare their predictive potential. IMs predicted low values in both, but lost their predictive power once the depressive brooding scale was added to predict depression/anxiety and well-being. The group of other AAs predicted depression/anxiety (positively), and maintained its predictive power when depressive brooding and the scale for repetitive thinking were added to the prediction. Thus, the group of other AAs predicted trait depression/anxiety over and above measures of rumination.

The loss of the predictive power of IM when entering rumination as predictor, is congruent with the negative correlation between IM and the two rumination scales. It probably reflects the shared reference of both constructs to the valence of an argument for the self. The group of other AAs, in contrast, did not correlate with ruminative or reflective dispositions, because they can be used for either purpose. Apparently, some general forms of reasoning about life are more frequent in trait depression/anxiety.

Finally, we had expected ruminators’ mood to improve less from narrating the five memories than the mood of reflectors. Although ruminators’ mood was significantly worse both before and after the study session than the mood of reflectors and of unconcerned, all participants’ mood improved equally over the course of the study session. This shows how narrating emotional, potentially troubling autobiographical memories to an attentive, understanding listener improves the immediate well-being of narrators, and thus the value of narrating as a cognitive-communicative format of processing experiences (Fioretti et al., 2017; Smorti, 2018; Wainryb et al., 2018). However, this study does not support the contention that habitual rumination impedes this kind of profiting from narrating problematic personal experiences.

Another point of interest is the limited correlation between IMs and indicators of AR. The correlation between IM and other AAs was small, and they did not correlate at all with person-event-connections and changes of views. However, small correlations with the change-relatedness of self-event connections (but not of changes of views) and with the positivity of changes of views confirm that IMs capture aspects of change-related, positively evaluated AR.

One might question the degree to which the narratives actually reflect ruminative tendencies, first because this kind of narrating is intentional and thus differs from the involuntary character of rumination, and second one might have expected that narratives by ruminators would be longer due to the inconclusive nature of rumination, but they were not. However, if the narrative elicitation instruction reminded participants of an event they tended to ruminate about and with which they had not yet come to terms, then ruminative processing might be expected to show when narrating, because narrating requires a sequential organization with a conclusion. One possible factor is the social nature of narrating, which might inhibit open rumination due to shame.

Another problem results from creating extreme groups on the basis of scales for dispositional rumination and reflection because they tend to correlate with each other. In this study we tried to reduce the problem by using two scales that did not measure depressive contents and therefore correlated less with each other. Still, the reflection scale was less than perfect because it neither directly addressed thinking about oneself nor the process qualities of reflective thinking such as intentionality, flexibility, or conclusiveness. Relatedly, we had not formulated any expectations for the unconcerned group that tended not to think too much about past experiences. They seemed to be, globally speaking, happier than both ruminators and reflectors judging by depression/anxiety and life satisfaction scores, although they narrated comparably negative experiences as indicated by the ratings.

### Limitations

This study was based on a non-clinical sample in which overall ruminative tendencies were weak. Possibly a clinical sample would have produced stronger differences between ruminators and reflectors. The groups were selected on the basis of self-report scales for the frequency of ruminative versus reflective thinking, which limits the grouping to self-reported thinking frequencies; in addition, the two scales correlated positively with each other, so that the differentiation between ruminative and reflective tendencies was less than perfect. The creation of extreme groups was only partially successful, inasmuch rumination and reflection values varied greatly within groups, because they differed only by having values above or below the mean on each scale plus by a defined difference between the standardized scores of both scales. This may have made it more difficult to detect group differences, but speaks for the validity of the differences that we did find. Also, the relative closeness of the two groups makes it less probable that the group which we did not study, people high both in trait rumination and trait reflection, would show different correlations between the variables. Finally, the group of unconcerned was more difficult to motivate to participate and therefore more self-selected and smaller than the other two groups of interest; therefore, their results need to be interpreted with caution.

### Implications and Future Directions

The study shows that IMs, a construct for change-oriented reasoning processes in psychotherapy, can also be found in autobiographical narratives outside the context of psychotherapy, where they also relate to measures of symptom levels and well-being in a way consistent with the construct. One group of indicators of AR, but not others, correlated positively with trait depression/anxiety, but not with rumination and reflection.

This finding points to two important research questions. First, we assumed, but did not actually code in the narratives whether ruminative reasoning processes manifest not only in thinking, but also in speaking and narrating. To date, two studies attempted to operationalize ruminative thinking in narratives. Whereas Marin and Rotondo’s (2017) coding of rumination in narratives was somewhat similar to IMs by identifying negative versus positive statements about the self, the coding by Hoyt et al. (2016) focused on more specific contents beyond valence. They coded written texts about one’s deepest emotions and imagined goal attainment for worrying, depressive, or angry thoughts as well as for planning, value clarification, goal-focused reflection, and discovery of meaning. They were able to predict health care visits 2 years later.

However, neither of these two studies had coded the defining formal aspects of ruminative thinking such as circularity, abstractness, lack of relevance to the situation at hand, and inconclusiveness. Clinical experience suggests that they may show in speaking and narrating. They might manifest in the absence of the actual narrating of event sequences, in the posing of question that do not lead to answers, in the repetition of arguments or statements, and in the inconclusiveness of narratives.

Second, with regard to AAs, it remains an open question which specific ways of using them reflects levels of depressive and anxiety symptoms or lack of well-being, and which are helpful in processing a negative experience. Like several earlier studies, we confirmed that a negative valence of AAs reflects less well-being. In contrast to clinical findings by Huang et al. (2020), the change-relatedness of AAs was not related to well-being and symptoms. We suggest to further pursue the question of helpful and unhelpful uses of AAs by, for example, coding whether they are repeated, specific and convincing, and help coming to a conclusion.

## Data Availability Statement

The datasets presented in this study can be found in online repositories. The names of the repository/repositories and accession number(s) can be found below: psycharchives, http://dx.doi.org/10.23668/psycharchives.4562.

## Ethics Statement

The studies involving human participants were reviewed and approved by the Ethics Committee of the Faculty for Psychology and Sports Sciences of the Goethe University Frankfurt. The patients/participants provided their written informed consent to participate in this study.

## Author Contributions

TH designed the study with the help of all involved. ID, PE, SG, and CK collected and transcribed the data. CK, AL, and MK coded the autobiographical arguments. PE and SG coded the innovative moments, trained and supervised by JB. TH wrote the manuscript. MG, JB, and PE revised the manuscript. All authors contributed to the article and approved the submitted version.

## Conflict of Interest

The authors declare that the research was conducted in the absence of any commercial or financial relationships that could be construed as a potential conflict of interest.

## Footnotes

1 https://www.psychologie.hhu.de/arbeitsgruppen/allgemeine-psychologie-und-arbeitspsychologie/gpower.html
