# Supplementary material for: Differences Between Subclinical Ruminators and Reflectors in Narrating Autobiographical Memories: Innovative Moments and Autobiographical Reasoning
Source: Front Psychol. 2021 Mar 2;12:624644. doi: 10.3389/fpsyg.2021.624644 (PMC7982801; doi:10.3389/fpsyg.2021.624644)
Supplement: Supplementary file 1 [file Data_Sheet_1.docx]

**Supplementary material**

**Narrative instructions [Translated from German – original available from first author]**

I will begin by telling you what to expect. The interview has two parts. First, I will ask you for five different experiences. After each of the five questions I ask you to select one experience and to write it down with some words. Please choose a specific event, that is an event that ideally took place on a specific day. This event should have happened at least half a year ago and at the most five years ago. What you wrote down will serve you as a memory aid and will not be used by us. Once you have selected and written down the five events, I will ask you to narrate each of the events. Do you have any further questions?

1. Selection of events

Please first consider, which turning points you have experienced in the course of your life. Turning points are experiences that changed the course of your life by changing your life circumstances or your way of thinking, feeling, or acting. Please choose and write down a turning point experience that is specific – that is an event that possibly took place on a specific day – and that to you is an especially important turning point in your life. [If participant cannot think of an event:] Maybe it helps to think of an event that changed how you think of certain things. [If consideration takes several minutes:] If you find it difficult to decide which event to choose, just take the one that came to your mind first. [Participant writes down an event]

Now please choose a specific event in which you were disappointed by yourself. [If consideration takes several minutes:] If you find it difficult to decide which event to choose, just take the one that came to your mind first. [Participant writes down an event]

Next please choose an experience in which you hurt someone or really made someone suffer. [If participant cannot think of an event:] Maybe an experience comes to your mind when someone felt hurt by you? [If consideration takes several minutes:] If you find it difficult to decide which event to choose, just take the one that came to your mind first. [Participant writes down an event]

Next please choose an experience of having been rejected or when someone left you. [If participant cannot think of an event:] If you have never been left by someone, please think of a time you were rejected. [If consideration takes several minutes:] If you find it difficult to decide which event to choose, just take the one that came to your mind first. [Participant writes down an event]

Finally, please choose an experience from which you learned an important lesson for life. [If participant cannot think of an event:] Maybe you can think of an experience that made you change how you act in specific situations. [If consideration takes several minutes:] If you find it difficult to decide which event to choose, just take the one that came to your mind first. [Participant writes down an event]

2. Narrating

Now comes the second part. I will ask you to narrate each of the five experiences. Please narrate so that it will become clear to me what actually happened and why this was relevant for you. Please also tell me, what this experience tells me abut you and how it influenced you in your personal development.

Let us start with the turning point experience. Please narrate all the events that took place on that day, how you experienced them, and which thoughts and feelings you had then, and which thoughts and feelings you have when looking back. [Participant narrates. If participant does not narrate a specific event:] Please think again, whether you can think of a very specific turning point experience that happened on one specific day. [If participant does not narrate but only summarizes event:] You have just summarized what happened back then. I would like to ask you to narrate the events so that I can really imagine all the things that happened. What exactly happened, event after event? What had you been thinking, what did you feel? [Standard probes always asked after the narration:] Please tell me, why exactly this was a turning point for you and what it tells me about you as a person. What were the consequences for you and your life? How did it change you and your life? [If participant does not explicate turning point for self:] Please think a moment how this was a turning point for yourself. How did it change you and your way of acting? [equivalent questions were asked for the following four events]
